# Computed Tomography Findings in a Case of Uterine Rupture as a Complication of Pyometra

**DOI:** 10.7759/cureus.53154

**Published:** 2024-01-29

**Authors:** Fadhila Mohd Hanapiah, Zul Khairul Azwadi Ismail, Othman Puteh, Mohd Ezane Aziz

**Affiliations:** 1 Radiology, Universiti Sains Malaysia School of Medical Sciences, Kubang Kerian, MYS

**Keywords:** pneumoretroperitoneum, pneumoperitoneum, pyometra rupture, uterine rupture, pyometra

## Abstract

Uterine rupture in the setting of pyometra is a rare occasion, with an incidence of less than 0.5%. The clinical manifestation of a perforated pyometra is non-specific; therefore, it can mimic many other causes of acute abdomen, such as perforated viscus, acute appendicitis, or diverticulitis, which poses unique challenges to diagnosis solely based on clinical information. We reviewed a case of an elderly postmenopausal lady who presented with a sudden onset of generalized abdominal pain, preceded by fever and vomiting. Physical examination revealed a distended abdomen with clinical signs of peritonism. She was initially diagnosed with possible obstructed gastrointestinal carcinoma by clinical examination, with the differential diagnosis of diverticular abscess. Eventually, further abdominal and pelvic contrast-enhanced computed tomography (CECT) study revealed a pyometra with uterine rupture, complicated with pneumoretroperitoneum and pneumoperitoneum. This case emphasizes the value of a CT scan in establishing an accurate diagnosis and early detection of life-threatening complications, such as uterine rupture, as in this case.

## Introduction

Pyometra refers to a collection of pus within the uterine cavity. Spontaneous rupture of pyometra is a rare occurrence, with an incidence of only 0.01 - 0.05% [1] in patients presented with gynaecological causes of abdominal pain. It has been estimated that mortality from spontaneous perforation of pyometra is more than 40%. It is commonly associated with pre-existing uterine diseases ranging from benign causes to gynaecological malignancy. The clinical manifestation of a perforated pyometra is non-specific and may mimic any other causes of an acute abdomen with peritonism such as perforated viscus, acute appendicitis, or diverticulitis, which poses unique challenges to diagnosis solely based on clinical information.

Ultrasonography is the first-line imaging modality for the evaluation of pyometra. However, it has a limited role in cases of perforation due to the presence of air casting acoustic shadowing on the deeper pelvic or abdominal structures. Therefore, further assessment with a cross-sectional imaging modality, such as CT scan, is required. Fast and accurate pre-operative diagnosis of perforated pyometra is essential for surgical planning and early intervention to reduce morbidity and mortality. To the best of our knowledge, several cases of perforated pyometra have been reported in the English literature, but only a few were diagnosed pre-operatively [1,2,3]. Here, we report a very rare case of uterine rupture secondary to pyometra that was diagnosed pre-operatively by a CT scan imaging.

## Case presentation

A 75-year-old postmenopausal lady presented to the emergency department with an acute onset of generalized abdominal pain, preceded by fever for 2 days and associated with vomiting. The pain was dull aching in nature, and aggravated by movement. The patient claimed she was still able to pass flatus, had no episode of altered bowel habits, and denied any family history of malignancy. She was medically treated for bronchial asthma, hypertension, and hyperlipidaemia, with no history of surgery. On clinical examination, the abdomen was distended with generalized tenderness and guarding. A vague mass was palpable in the lower abdomen. She appeared ill-looking with vital signs as follows: blood pressure 108/63 mmHg, heart rate 115 beats/min, oxygen saturation 97% under room air, and a documented body temperature of 39^o^C. The initial blood investigation revealed a high total white count of 29x10^9^/L. She was put on 3 pints of normal saline for IV drip maintenance and started on a combination of intravenous antibiotics cefoperazone and metronidazole. The case was initially referred to the surgical team with the diagnosis of a possible gastrointestinal malignancy with partially obstructed sigmoid carcinoma with the differential diagnosis of diverticulitis. The patient was admitted to the surgical ward; however, her condition deteriorated with respiratory distress, requiring ventilatory support in the ICU. The provisional diagnosis at that time was sepsis secondary to intra-abdominal collection. The case was then referred to the obstetrics and gynaecology (O&G) team to rule out any gynaecological causes. 

**Figure 1:**
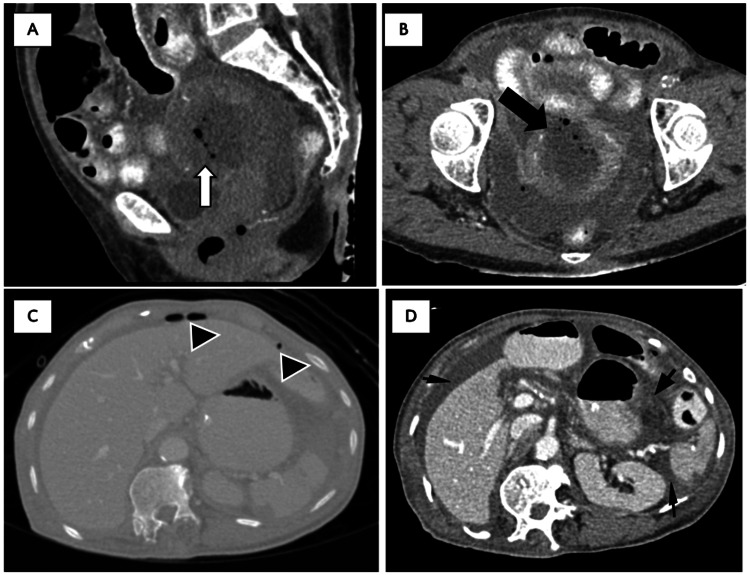
Contrasted CT images Sagittal reformatted CECT scan of the abdomen and pelvis showed an ill-defined hypodense collection (white arrow) within the uterine cavity with multiple air pockets (A), axial sections of the pelvis demonstrated focal uterine wall discontinuity (black arrow) at the fundus consistent with rupture (B), presence of pneumoperitoneum (arrowhead) (C) and intra-abdominal free-fluid (thin arrow) (D).

Trans-abdominal ultrasound (TAS) assessment demonstrated a bulky uterus with irregular endometrial lining and intra-uterine collection. Free fluid was also seen surrounding the uterus. A CT scan of the abdomen and pelvis (refer Figure 1) revealed a distorted uterus and the presence of an ill-defined hypodense collection within the uterine cavity with bilateral parametrial fat stranding. There were multiple air pockets within the collection and foci of calcification within the uterine wall. There was a focal area of discontinuity at the uterine fundus in keeping with uterine rupture. Pockets of air and fluid collection with density similar to the intrauterine collection were seen within the pelvic cul-de-sac, which further confirmed the presence of a perforation. The CT scan also showed findings of pneumoperitoneum and pneumoretroperitoneum, which could indicate disruption of the peritoneal lining adjacent to the uterus. Moderate ascites was also observed.

A total abdominal hysterectomy with bilateral salpingo-oophorectomy (TAHBSO) was performed. The intraoperative findings revealed gross peritoneal contamination involving both subphrenic spaces, subhepatic space, bilateral paracolic gutters, and pelvic cavity. Slough and pus were seen disseminated all over the peritoneal cavity with associated interloop collection. An area of perforation was observed at the uterine fundus, measuring about 1.0cm x 2.0cm, with discharging pus noted through the opening. The uterus was then cut open, disclosing foul-smelling pus and necrotic tissues within the endometrial cavity, which were then evacuated. A total of 600cc of pus was removed from the peritoneal and endometrial cavities followed by peritoneal lavage with copious amounts of saline, amounting to 12 litres. Histopathological study revealed uterine abscess, with no evidence of malignancy. However, pus culture and sensitivity taken identified *Escherichia coli* within the sample.

The surgery had an excellent outcome and the patient was able to gradually recover from septicaemia. She spent almost 3 weeks in the ICU, and then was allowed to be transferred to the general ward. She was discharged on postoperative day 52 with only 1 complication of surgical wound breakdown. 

## Discussion

Pyometra, or the collection of purulent material within the uterine cavity, is essentially an extremely rare condition. It is often associated with cervical stenosis or blockage, preventing the natural drainage of uterine secretion [4]. It is typically associated with postmenopausal women or those with underlying uterine malignancy in most of the cases. Several other aetiologies include endometritis, pelvic inflammatory disease, prior cervical surgery, pelvic irradiation, or retained intra-uterine contraceptive device (IUCD). However, in our case, no definite identifiable cause is found. There was no evidence of malignancy intra-operatively, and she had no prior endometrial biopsy or dilatation curettage procedure. 

Nevertheless, the most probable cause might be due to post-menopausal changes and cervical stenosis causing degenerative changes of the uterine wall [5], characterized by sloughing of the uterine wall that leads to stagnation of discharge, resulting in anaerobic infection causing perforation at the fundus. Commonly isolated organisms from pyometras include* Streptococcus* species, *Bacteroides fragilis*, and *Escherichia coli *[6,7]. In our case, *E. coli* has been identified as the culprit. *E. coli* is a normal intestinal commensal that can also colonize the vaginal microbiota. It can also be highly pathogenic to the reproductive system when present in abundance. It is a highly virulent micro-organism that can cause endometritis, septicaemia, and uterine necrosis, which necessitates definitive surgical intervention to achieve the resolution of the infection. The organism likely has caused inflammation and weakening of the uterine tissues. As the integrity of the uterine wall has been affected, the potential risk of rupture is inevitable.

## Conclusions

The clinical findings of perforated pyometra usually mimic gastrointestinal tract perforation, posing challenges to clinicians to arrive at an accurate diagnosis pre-operatively. Conventionally, the diagnosis is reached by exploratory laparotomy. The role of transabdominal ultrasound is limited due to the presence of air, and it has low sensitivity to detect small perforations. CT scan is indispensable in reaching an accurate diagnosis and location of the perforation by exhibiting a focal area of uterine wall discontinuity. Although spontaneously perforated pyometra is a rare entity, the condition must be considered when dealing with postmenopausal women with acute abdominal pain with clinical manifestations of peritonitis. Awareness of risk factors can contribute to the prevention and early detection of conditions that may lead to pyometra in order to mitigate the risk of uterine rupture. A prompt diagnosis with early intervention is proven to save lives from this catastrophic complication of pyometra.
